# Evaluation of the Association Between Maternal Anemia, Oral Mucosal Changes, and Adverse Birth Outcomes Among Pregnant Women

**DOI:** 10.7759/cureus.111118

**Published:** 2026-06-18

**Authors:** Sameer Chauhan, Honey Sharma, Riny George, Heena Dixit, Akriti Mahajan, Afroz K Syed

**Affiliations:** 1 Department of Prosthodontics, K. M. Shah Dental College and Hospital, Sumandeep Vidyapeeth (Deemed to be University), Vadodara, IND; 2 Department of Obstetrics and Gynecology, Sri Maharaja Gulab Singh (SMGS) Hospital, Jammu, IND; 3 Department of Oral and Maxillofacial Surgery, Al-Azhar Dental College, Thodupuzha, IND; 4 Department of Public Health, Commissionerate of Health and Family Welfare, Government of Telangana, Hyderabad, IND; 5 Department of Oral Medicine and Radiology, Desh Bhagat Dental College and Hospital, Mandi Gobindgarh, IND; 6 Deaprtment of Dentistry, Anne Yarlagadda Charitable Trust, Vijayawada, IND

**Keywords:** anemia, low birth weight, oral mucosa, pregnancy, pregnancy outcomes, preterm birth, stillbirth

## Abstract

Introduction: Maternal anemia remains a major public health concern associated with adverse maternal and neonatal outcomes worldwide. Oral mucosal changes are also commonly observed in anemic individuals and may reflect the systemic severity of nutritional and hematological deficiencies. This study aimed to evaluate the association between maternal anemia, oral mucosal changes, and adverse birth outcomes among pregnant women.

Materials and methods: This prospective observational cohort study was conducted among 174 pregnant women attending antenatal clinics at Desh Bhagat Dental College and Hospital (Mandi Gobindgarh, PB, IND) in association with the Department of Obstetrics and Gynecology of the affiliated teaching hospital. Hemoglobin (Hb) levels were estimated during the first trimester using an automated hematology analyzer, and comprehensive oral examinations were performed to assess oral mucosal changes associated with anemia. Participants were followed until delivery, and birth outcomes, including low birth weight, preterm birth, and stillbirth, were recorded. Statistical analysis was performed using an independent t-test, chi-square test, relative risk analysis, and multivariable logistic regression. Statistical significance was set at p < 0.05.

Results: Among the 174 enrolled women, 72 (41.4%) were anemic, and 102 (58.6%) were non-anemic. Mild anemia was observed in 31 (17.8%), moderate anemia in 34 (19.5%), and severe anemia in seven (4.0%) participants. Oral mucosal changes were significantly more prevalent among anemic women than among non-anemic women (p < 0.001). Low birth weight was significantly higher among women with anemia (p < 0.001), while preterm birth occurred in 24 (33.3%) anemic women compared with 14 (13.7%) non-anemic women (p = 0.002). Stillbirth was observed in seven (9.7%) anemic participants and two (2.0%) non-anemic participants (p = 0.016). Oral mucosal changes were independently associated with low birth weight, whereas maternal anemia remained an independent predictor of low birth weight.

Conclusion: Maternal anemia was significantly associated with oral mucosal changes and adverse birth outcomes, particularly low birth weight, preterm birth, and stillbirth. Increasing anemia severity demonstrated a dose-dependent relationship with worsening oral and neonatal outcomes. Early antenatal hematological and oral screening, along with timely nutritional intervention and appropriate management of anemia, may help improve maternal and neonatal health outcomes.

## Introduction

Anemia during pregnancy remains one of the most common nutritional and hematological disorders affecting women worldwide, particularly in low- and middle-income countries [1]. Studies conducted in high-risk populations have reported that the overall prevalence of anemia may range from 50% to 80%, whereas approximately 10% to 20% of individuals may present with moderate-to-severe forms of anemia [2]. The burden of anemia is particularly greater among individuals belonging to lower socioeconomic groups, those with low body weight, and women with a recent history of childbirth [3]. Physiological changes during pregnancy increase iron requirements, and inadequate nutritional intake, poor socioeconomic conditions, infections, and limited access to antenatal care further contribute to the burden of maternal anemia [4].

Maternal anemia is characterized by reduced hemoglobin (Hb) concentration, which impairs oxygen transport to the maternal and fetal tissues. This reduction in oxygen-carrying capacity may adversely affect placental function and fetal growth, leading to unfavorable pregnancy outcomes [5]. Previous studies have demonstrated that anemia during pregnancy is associated with an increased risk of low birth weight, preterm birth, intrauterine growth restriction, perinatal mortality, and stillbirth [5,6]. Severe anemia has also been linked to maternal complications such as fatigue, infections, postpartum hemorrhage, and an increased risk of maternal mortality [7].

Despite advances in antenatal healthcare and iron supplementation programs, the prevalence of anemia during pregnancy remains high in many developing regions [3]. Furthermore, variations in socioeconomic status, nutritional practices, parity, maternal age, and access to healthcare services may influence the occurrence of anemia and its associated outcomes. Although several studies have investigated the relationship between maternal anemia and adverse birth outcomes, inconsistent findings and regional variations highlight the need for further prospective evidence [4-6].

Maternal anemia may also manifest clinically within the oral cavity because of impaired oxygen delivery, nutritional deficiencies, and altered epithelial metabolism. Common oral manifestations associated with anemia include pallor of the oral mucosa, atrophic glossitis, angular cheilitis, depapillation of the tongue, oral candidiasis, burning sensation of the oral mucosa, and recurrent aphthous ulceration [8]. These lesions may adversely affect oral comfort, nutrition, and quality of life and can serve as early clinical indicators of underlying hematological deficiency. Previous studies have suggested that oral mucosal changes are more prevalent among individuals with moderate-to-severe anemia; however, limited prospective evidence is available regarding their occurrence during pregnancy and their potential association with adverse birth outcomes [8,9]. Therefore, evaluation of oral manifestations alongside hematological and obstetric parameters may provide additional insight into the systemic burden and clinical impact of maternal anemia.

Therefore, the present prospective observational cohort study was designed to evaluate the association between maternal anemia, oral mucosal changes, and adverse birth outcomes among pregnant women. The study aimed to assess the prevalence and severity of anemia, evaluate the pattern of oral mucosal manifestations associated with anemia, and determine their relationship with adverse pregnancy outcomes, including low birth weight, preterm birth, and stillbirth. Additionally, the influence of maternal demographic and clinical factors on oral and obstetric outcomes was analyzed. Hemoglobin estimation and oral examinations were performed at enrollment during early pregnancy, and participants were followed until delivery, with low birth weight considered the primary outcome and oral mucosal changes, preterm birth, and stillbirth considered secondary outcomes.

## Materials and methods

Study design, setting, duration, and ethical approval

This prospective observational cohort study was primarily conducted by the Department of Oral Medicine and Radiology, Desh Bhagat Dental College and Hospital (Mandi Gobindgarh, PB, IND), in association with the Department of Obstetrics and Gynecology of the Desh Bhagat Hospital. The study was conducted over a total duration of 24 months from April 2023 to April 2025, which included participant recruitment, outcome assessment, data collection, and data analysis. Participant recruitment was carried out from April 2023 to March 2024, and all enrolled participants were prospectively followed until delivery and completion of outcome assessment. Pregnant women attending the antenatal outpatient department during their first antenatal visit were recruited and prospectively followed until delivery to evaluate the association between maternal anemia and adverse birth outcomes.

The study protocol was reviewed and approved by the Institutional Ethical Committee (approval no. DBDCH/IEC/2023/04/029). This study was conducted in accordance with the ethical principles outlined in the Declaration of Helsinki. Written informed consent was obtained from all participants after explaining the nature, objectives, benefits, and potential risks of the study in their local language. Confidentiality of participant information was strictly maintained throughout the study, and participation was voluntary. The participants were assured that their refusal to participate or withdraw from the study at any stage would not affect their routine antenatal care or treatment.

Eligibility criteria

Pregnant women aged 18 years and above with singleton pregnancies and a gestational age of 14 weeks or less, as confirmed by first-trimester ultrasonography, were considered eligible for inclusion in the study. Only women willing to undergo hematological investigations and complete antenatal follow-up until delivery were included. Participants were also required to reside within the hospital catchment area to ensure adequate follow-up and outcome assessment. Women with multiple gestations, including twins or triplets, were excluded from the study. Pregnant women with known hematological or systemic disorders affecting Hb levels, such as sickle cell disease, thalassemia major, chronic kidney disease, malignancy, or active bleeding disorders, were also excluded. Participants with a history of blood transfusion within the preceding three months, prior use of iron supplements or erythropoiesis-stimulating agents before enrollment, and those who experienced miscarriage or medical termination of pregnancy before 20 weeks of gestation were excluded from the final analysis.

Sample size calculation

Sample size was calculated using G*Power (Heinrich Heine University, Düsseldorf, DEU). The calculation assumed an anemia prevalence of approximately 40%, a low birth weight prevalence of 10% among non-anemic women, and an anticipated relative risk of 2.0 for low birth weight among anemic women [10]. Considering a study power of 80% and a two-sided alpha error of 5%, the minimum required sample size was estimated to be 148 pregnant women (74 per group). To compensate for a possible 15% loss to follow-up due to miscarriage, transfer, or incomplete follow-up, the final sample size was increased to 174 patients.

Study methodology

Eligible pregnant women who attended the antenatal outpatient clinic during their first trimester were screened and consecutively recruited after obtaining informed consent. At enrollment, detailed demographic and clinical information was obtained using a structured questionnaire. Information regarding maternal age, parity, educational status, socioeconomic status, dietary habits, medical history, obstetric history, and medication use was recorded. Anthropometric measurements, including height and weight, were obtained using standardized instruments, and BMI was calculated as weight in kilograms divided by height in meters squared.

A comprehensive intraoral examination was performed at baseline by a trained dental specialist under adequate illumination using sterile mouth mirrors and gauze. Oral mucosal changes associated with anemia were assessed and documented using a standardized clinical examination protocol. The oral manifestations evaluated included mucosal pallor, atrophic glossitis, angular cheilitis, recurrent aphthous ulceration, depapillation of the tongue, burning sensation of the oral mucosa, and oral candidiasis [8,9]. Mucosal pallor was identified as generalized blanching of the oral mucosa, angular cheilitis as erythematous fissuring at the labial commissures, glossitis as depapillation or atrophy of the dorsal tongue, and oral candidiasis as clinically evident pseudomembranous or erythematous lesions. The presence or absence of each lesion was recorded. To minimize inter-examiner variability, all examinations were conducted by a single examiner throughout the study. Participants requiring dental or oral medical care were referred for appropriate management without affecting their inclusion in the study.

Venous blood samples (5 mL) were collected under aseptic conditions in ethylenediaminetetraacetic acid (EDTA) tubes for hematological analysis. Complete blood counts were performed using an automated hematology analyzer (Sysmex XN-1000, Sysmex Corp., Kobe, Japan). The laboratory personnel who performed the investigations were blinded to the participants’ clinical details. Maternal anemia was defined as a Hb concentration less than 11 g/dL during pregnancy. Anemia severity was further categorized as mild anemia (10.0-10.9 g/dL), moderate anemia (7.0-9.9 g/dL), and severe anemia (<7.0 g/dL) [9].

The participants were followed up monthly according to routine antenatal care schedules. Compliance with iron and folic acid supplementation prescribed during antenatal visits was assessed during follow-up through self-reporting and review of remaining tablets brought by participants. Repeated Hb estimation was performed during 28-32 weeks of gestation to monitor maternal hematological status during pregnancy. Participants with incomplete follow-up or pregnancy loss before 20 weeks of gestation were excluded from the final analysis.

Delivery and neonatal outcome data were collected from hospital records immediately after childbirth by trained obstetricians and pediatric healthcare personnel who were blinded to maternal anemia status. The primary birth outcomes assessed were low birth weight, preterm birth, and stillbirth. Low birth weight was defined as a birth weight of less than 2500 g, measured using a calibrated digital neonatal weighing scale [11]. Preterm birth was defined as delivery before the completion of 37 weeks of gestation based on the last menstrual period confirmed by first-trimester ultrasonography [12]. Stillbirth was defined as fetal death occurring at or after 20 weeks' gestation [13]. In addition to hematological and obstetric parameters, oral mucosal findings were considered secondary outcome variables. The prevalence and pattern of oral mucosal changes among anemic and non-anemic pregnant women were evaluated, and their association with anemia severity was analyzed.

Statistical analysis

Statistical analyses were performed using SPSS Statistics version 25 (IBM Corp., Armonk, NY, USA). Normality of data distribution was assessed using the Shapiro-Wilk test. Continuous variables are expressed as mean ± standard deviation, whereas categorical variables are presented as frequencies and percentages. An independent-samples t-test was used to compare continuous variables, whereas the Pearson chi-square test was used to compare categorical variables. The relative risk (RR) with a 95% CI was calculated for adverse oral mucosal lesions and birth outcomes, including low birth weight, preterm birth, and stillbirth. Multivariable logistic regression analysis was performed to determine the independent predictors of low birth weight and oral mucosal lesions after adjusting for potential confounding variables, including maternal age, parity, BMI, socioeconomic status, gestational age at enrollment, comorbidities, and adherence to iron supplementation. All covariates were entered simultaneously into the regression model based on their clinical relevance and potential association with maternal anemia and pregnancy outcomes. Adjusted odds ratios (aORs) with 95% CIs are reported. Statistical significance was set at p < 0.05.

## Results

A total of 174 pregnant women were enrolled, of whom 72 (41.4%) were anemic, and 102 (58.6%) were non-anemic. Nulliparity was significantly more frequent among anemic women (p = 0.028). Lower socioeconomic status was more prevalent in the anemic group (p = 0.002), while iron/folic acid supplementation adherence was lower (p = 0.005). Comorbidity was more common among anemic women (p = 0.018). Oral mucosal changes were markedly more prevalent in the anemic group (p < 0.001), indicating a strong association between anemia and oral mucosal pathology at baseline (Table 1).

**Table 1:** Baseline sociodemographic and clinical characteristics according to anemia status Data presented as mean ± standard deviation or n (%); independent samples t-test used for continuous variables; Pearson chi-square test used for categorical variables; *p < 0.05 considered statistically significant

Characteristic		Total (n = 174)	Anemic (n = 72)	Non-anemic (n = 102)	Test statistic	p-value
Maternal age (years)	Mean ± SD	24.8 ± 4.6	23.9 ± 4.2	25.4 ± 4.8	t = −2.13	0.034*
Parity	Nulliparous	89 (51.1%)	44 (61.1%)	45 (44.1%)	χ² = 4.82	0.028*
	Multiparous	85 (48.9%)	28 (38.9%	57 (55.9%)		
BMI at enrollment (kg/m²)	Mean ± SD	22.3 ± 3.1	21.4 ± 2.8	23.0 ± 3.2	t = −3.29	0.001*
Socioeconomic status	Upper/upper-middle	41 (23.6%)	9 (12.5%)	32 (31.4%)	χ² = 12.47	0.002*
	Lower-middle	88 (50.6%)	38 (52.8%)	50 (49.0%)		
	Lower	45 (25.9%)	25 (34.7%)	20 (19.6%)		
Gestational age at enrollment (weeks)	Mean ± SD	11.2 ± 1.8	11.0 ± 1.7	11.4 ± 1.9	t = −1.38	0.169
Iron/folic acid supplementation (adherent)	Yes	119 (68.4%)	41 (56.9%)	78 (76.5%)	χ² = 7.94	0.005*
	No	55 (31.6%)	31 (43.1%)	24 (23.5%)		
Comorbidity (any)	Present	38 (21.8%)	22 (30.6%)	16 (15.7%)	χ² = 5.60	0.018*
	Absent	136 (78.2%)	50 (69.4%)	86 (84.3%)		
Oral mucosal changes	Present	48 (27.6%)	42 (58.3%)	6 (5.9%)	χ² = 62.14	< 0.001*
	Absent	126 (72.4%)	30 (41.7%)	96 (94.1%)		

Among the 72 (41.4%) anemic women, mild anemia accounted for 31 (17.8%), moderate anemia for 34 (19.5%), and severe anemia for seven (4.0%). Oral mucosal changes were absent in the majority of non-anemic women, with only six (5.9%) affected. In contrast, mucosal changes were found in 13 (41.9%) of those with mild anemia, rising sharply to 22 (64.7%) in moderate anemia, and affecting all seven (100.0%) women with severe anemia. The predominant mucosal finding progressed from pallor of mucosa in mild cases to angular cheilitis in moderate cases and glossitis or atrophic tongue in severe anemia, reflecting escalating mucosal pathology with worsening Hb deficit (Table 2).

**Table 2:** Distribution of Hb levels and oral mucosal changes among the participants Data presented as mean ± SD or n (%); Hb was measured at enrollment during gestational age ≤14 weeks using an automated complete blood count analyzer; 95% CI calculated for mean Hb levels Hb: Hemoglobin

Anemia category	n (%)	Mean Hb (g/dL) ± SD	95% CI	Oral mucosal changes n (%)	Most common mucosal finding
Non-anemic (Hb ≥ 11.0 g/dL)	102 (58.6%)	12.3 ± 0.8	12.1-12.5	6 (5.9%)	Mild pallor
Anemic (Hb < 11.0 g/dL) - Total	72 (41.4%)	9.2 ± 1.4	8.9-9.5	42 (58.3%)	Changes in mucosa (pallor and atrophy)
Mild anemia (Hb 10.0-10.9 g/dL)	31 (17.8%)	10.4 ± 0.3	10.3-10.5	13 (41.9%)	Pallor of mucosa
Moderate anemia (Hb 7.0-9.9 g/dL)	34 (19.5%)	8.6 ± 0.8	8.3-8.9	22 (64.7%)	Angular cheilitis
Severe anemia (Hb < 7.0 g/dL)	7 (4.0%)	6.1 ± 0.5	5.7-6.5	7 (100.0%)	Glossitis/atrophic tongue

Low birth weight occurred in 49 (28.2%) of all participants, being significantly more frequent among anemic women (p < 0.001). Among women with oral mucosal changes, low birth weight was recorded in 29 (60.4%). Preterm birth affected 38 (21.8%) overall, with a higher burden in the anemic group (p = 0.002); 21 (43.8%) of those with mucosal changes delivered preterm. Stillbirth occurred in nine (5.2%) women, predominantly among anemic participants (p = 0.016), with seven (14.6%) stillbirths recorded in the oral mucosal changes subgroup (Table 3).

**Table 3:** Incidence of adverse birth outcomes according to maternal anemia status Data presented as n (%); Pearson chi-square test was used for comparison between groups; multivariable logistic regression was used to calculate aOR; *p < 0.05 considered statistically significant RR: Relative risk, aOR: Adjusted odds ratios

Birth outcome	Total (n = 174)	Anemic (n = 72)	Non-anemic (n = 102)	χ²	RR (95% CI)	aOR (95% CI)	p-value
Low birth weight (<2500 g)	49 (28.2%)	31 (43.1%)	18 (17.6%)	13.82	2.44 (1.54–3.87)	2.61 (1.43–4.76)	< 0.001*
Preterm birth (<37 weeks)	38 (21.8%)	24 (33.3%)	14 (13.7%)	9.57	2.43 (1.40–4.21)	2.18 (1.12–4.24)	0.002*
Stillbirth (≥20 weeks)	9 (5.2%)	7 (9.7%)	2 (2.0%)	5.81	4.96 (1.08–22.7)	4.02 (0.79–20.5)	0.016*

A significant dose-response relationship was observed across anemia severity categories. Low birth weight rose from 18 (17.6%) in non-anemic women to 10 (32.3%) in mild anemia, 16 (47.1%) in moderate anemia, and five (71.4%) in severe anemia. Oral mucosal changes followed the same gradient: six (5.9%), 13 (41.9%), 22 (64.7%), and seven (100.0%) across severity groups. The linear trend across ordered severity groups was statistically significant (p < 0.001), confirming a progressive escalation of both mucosal changes and birth complications with increasing anemia severity (Table 4).

**Table 4:** Dose-response relationship between anemia severity and adverse birth outcomes Data presented as n (%); Mantel-Haenszel chi-square test was used for trend analysis; multivariable logistic regression was performed using the non-anemic group as the reference category; *p < 0.05 considered statistically significant aOR: Adjusted odds ratio, Hb: Hemoglobin, df: Degree of freedom

Anemia severity	Total (n = 174)	Low birth weight, n (%)	Preterm birth, n (%)	Stillbirth, n (%)	Oral mucosal changes, n (%)	aOR for oral mucosal changes (95% CI)	aOR for low birth weight (95% CI)	p-value
Non-anemic (Ref)	102	18 (17.6%)	14 (13.7%)	2 (2.0%)	6 (5.9%)	1.00 (Reference)	1.00 (Reference)	—
Mild anemia (Hb 10.0-10.9)	31	10 (32.3%)	8 (25.8%)	1 (3.2%)	13 (41.9%)	1.67 (0.62-4.49)	1.93 (0.82-4.54)	0.132
Moderate anemia (Hb 7.0-9.9)	34	16 (47.1%)	12 (35.3%)	3 (8.8%)	22 (64.7%)	3.12 (1.28-7.61)	3.28 (1.38-7.78)	0.007*
Severe anemia (Hb < 7.0)	7	5 (71.4%)	4 (57.1%)	3 (42.9%)	7 (100%)	—	10.6 (1.80-62.3)	0.009*
Test for linear trend across ordered anemia severity groups: χ² = 18.43, df = 1, p < 0.001*; Mantel-Haenszel χ² = 17.91, p < 0.001*								

Multivariable logistic regression identified anemia and oral mucosal changes as the two strongest independent predictors of low birth weight. Anemia conferred an aOR of 2.61 (p < 0.001), while the presence of oral mucosal changes yielded an aOR of 3.14 (p = 0.002). Lower BMI was a significant protective predictor (aOR = 0.85; p = 0.017), and comorbidity was associated with increased risk; low socioeconomic status, non-adherence to iron supplementation, and maternal age did not reach statistical significance. The model demonstrated good calibration with an overall correct classification rate of 75.3% (Table 5).

**Table 5:** Multivariable logistic regression analysis for predictors of low birth weight Data presented as odds ratio with 95% CI; multivariable logistic regression analysis adjusted for all listed variables simultaneously; Wald chi-square test was used to determine significance; Hosmer-Lemeshow test was used for model calibration; *p < 0.05 considered statistically significant aOR: Adjusted odds ratio

Variable	Crude OR	95% CI	aOR*	95% CI	Wald χ²	p-value
Anemia (vs. non-anemic)	3.59	1.83-7.04	2.61	1.43-4.76	10.42	< 0.001*
Oral mucosal changes (present vs. absent)	4.18	1.97-8.87	3.14	1.42-6.93	9.87	0.002*
Nulliparous (vs. multiparous)	2.14	1.12-4.09	1.88	0.96-3.68	3.56	0.059
BMI (per 1 kg/m² increase)	0.82	0.73-0.92	0.85	0.75-0.97	5.73	0.017*
Low socioeconomic status (vs. upper/mid)	2.86	1.35-6.07	2.03	0.88-4.67	2.82	0.093
Iron supplement non-adherent (vs. adherent)	2.48	1.28-4.80	1.92	0.94-3.91	3.38	0.066
Comorbidity present (vs. absent)	3.12	1.47-6.63	2.27	1.01-5.12	3.97	0.046*
Maternal age (per year increase)	0.94	0.87-1.01	0.95	0.88-1.03	1.53	0.216
Model fit: Hosmer-Lemeshow χ² = 6.74, p = 0.565, Nagelkerke R² = 0.31, Overall correct classification: 74.1%						

Any oral mucosal change was present in 48 (27.6%) participants, predominantly among anemic women, and was significantly associated with low birth weight. Pallor of the oral mucosa, the most common finding in 38 (21.8%), was associated with low birth weight in 21 (55.3%) affected women (aOR = 2.87; p = 0.014). Angular cheilitis showed a stronger association with low birth weight (p = 0.012). Glossitis, or atrophic tongue, carried the highest low birth weight risk (p = 0.028). Candidiasis and xerostomia showed elevated aORs but did not reach statistical significance (p = 0.082 and p = 0.241, respectively) (Table 6).

**Table 6:** Association between specific oral mucosal changes and adverse birth outcomes among study participants Data are presented as n (%); aOR adjusted for anemia status, age, parity, BMI, socioeconomic status, supplementation adherence, and comorbidity; oral mucosal changes were assessed clinically at enrollment by a trained examiner; *p < 0.05 aOR: Adjusted odds ratio

Type of oral mucosal change	Total (n = 174)	Anemic (n = 72)	Non-anemic (n = 102)	Low birth weight, n (%)	Preterm birth, n (%)	aOR for low birth weight (95% CI)	p-value
Any oral mucosal change	48 (27.6%)	42 (58.3%)	6 (5.9%)	29 (60.4%)	21 (43.8%)	3.14 (1.42-6.93)	0.002*
Pallor of oral mucosa	38 (21.8%)	34 (47.2%)	4 (3.9%)	21 (55.3%)	15 (39.5%)	2.87 (1.24-6.63)	0.014*
Angular cheilitis	22 (12.6%)	20 (27.8%)	2 (2.0%)	14 (63.6%)	10 (45.5%)	3.42 (1.31-8.92)	0.012*
Glossitis/atrophic tongue	14 (8.0%)	13 (18.1%)	1 (1.0%)	10 (71.4%)	8 (57.1%)	4.61 (1.18-17.98)	0.028*
Candidiasis/stomatitis	9 (5.2%)	8 (11.1%)	1 (1.0%)	6 (66.7%)	5 (55.6%)	3.98 (0.84-18.8)	0.082
Xerostomia (dry mouth)	7 (4.0%)	6 (8.3%)	1 (1.0%)	4 (57.1%)	3 (42.9%)	2.74 (0.51-14.7)	0.241

## Discussion

This prospective observational cohort study evaluated the association between maternal anemia and adverse birth outcomes in pregnant women attending antenatal care services. The findings demonstrated that anemia during pregnancy was significantly associated with an increased risk of low birth weight, preterm birth, and stillbirth. Additionally, the severity of anemia showed a clear dose-response relationship with worsening pregnancy outcomes. These findings emphasize the substantial impact of maternal Hb status on fetal growth and perinatal health.

In the present study, the prevalence of anemia among pregnant women was 41.4%, which is consistent with reports from developing countries where maternal anemia remains a major public health concern. Similar prevalence rates have been reported by Bencaiova et al. [14] and Kalaivani [15], who observed that nutritional deficiencies, poor socioeconomic conditions, and inadequate antenatal care significantly contributed to the high burden of anemia during pregnancy. The relatively high prevalence observed in the current study may be attributed to nutritional insufficiency, poor compliance with iron supplementation, and the lower socioeconomic status of the participants.

The study also demonstrated that women with anemia had significantly lower maternal age and BMI than those without anemia. Lower socioeconomic status and poor adherence to iron and folic acid supplementation were significantly associated with anemia [16]. These findings are supported by previous studies that have shown that younger maternal age, poor maternal nutrition, and inadequate healthcare access increase the risk of anemia during pregnancy [17,18]. Women from lower socioeconomic groups may experience reduced dietary iron intake, poor health literacy, and limited access to antenatal nutritional counseling, thereby predisposing them to anemia [16].

A major finding of the present study was the significantly increased incidence of low birth weight among mothers with anemia. Anemic women demonstrated more than two-fold higher adjusted odds of delivering low-birth-weight infants than non-anemic women. Similar findings were reported by Rahman et al. [19] and Figueiredo et al. [11], who observed strong associations between maternal anemia and impaired fetal growth. Reduced maternal Hb concentration decreases oxygen-carrying capacity and compromises placental oxygen transfer, resulting in intrauterine growth restriction and reduced fetal weight [11]. Chronic maternal hypoxia may also impair placental vascularization and nutrient transport, thereby contributing to fetal growth restriction [4].

Preterm birth was also significantly more common among women with anemia in the current study. These findings are in agreement with those of studies conducted by Stephen et al. [12] and Kozuki et al. [20], who demonstrated that maternal anemia increases the risk of premature delivery. A possible biological explanation may involve chronic placental hypoxia, oxidative stress, and increased maternal susceptibility to infections, which may trigger inflammatory pathways associated with early uterine contractions and premature labor. Furthermore, iron deficiency may impair maternal immune function and increase the vulnerability to infections that can precipitate preterm births.

The present study also identified a significantly higher occurrence of stillbirths among mothers with anemia. Although the absolute number of stillbirths was relatively small, the risk was considerably higher in women with moderate and severe anemia. Similar associations have been reported in earlier epidemiological studies, suggesting that severe maternal anemia can compromise fetal oxygenation to a critical extent, resulting in fetal distress and intrauterine fetal demise [7,21]. Severe anemia may also increase the likelihood of placental insufficiency, hypertensive disorders, and maternal cardiovascular stress, all of which contribute to poor fetal survival [12].

An important observation in the present study was the dose-response relationship between anemia severity and adverse birth outcomes. Women with severe anemia had the highest prevalence of low birth weight, preterm birth, and stillbirths [10,11]. This trend supports the biological plausibility of the observed associations and strengthens the evidence that worsening maternal anemia progressively increases the fetal risk. Similar dose-dependent relationships have been reported by Allen [22], who emphasized that severe reductions in maternal Hb levels substantially impair fetal oxygen delivery and placental function.

Multivariable logistic regression analysis further demonstrated that maternal anemia remained an independent predictor of low birth weight even after adjustment for confounding variables. Additionally, low maternal BMI and the presence of comorbidities significantly contributed to adverse outcomes. These findings suggest that maternal anemia often coexists with nutritional deficiencies and systemic health conditions, which may collectively influence pregnancy outcomes [23]. Therefore, antenatal screening programs should not only focus on Hb estimation but should also include comprehensive nutritional and medical assessments of pregnant women.

An important finding of the present study was the significantly higher prevalence of oral mucosal changes among anemic pregnant women compared with non-anemic women. The frequency and severity of oral manifestations increased progressively with worsening anemia severity, with pallor of the oral mucosa being the most common finding in mild anemia, whereas angular cheilitis and glossitis were more frequently observed in moderate-to-severe anemia [8,9]. These findings are consistent with previous studies that report a strong association between iron deficiency anemia and oral mucosal lesions, including mucosal pallor, depapillation of the tongue, atrophic glossitis, candidiasis, and angular cheilitis [8,9]. Reduced Hb levels and associated nutritional deficiencies may impair epithelial maturation and decrease tissue oxygenation, thereby increasing susceptibility to mucosal atrophy and opportunistic infections [24]. Furthermore, the present study demonstrated that oral mucosal changes were independently associated with an increased risk of low birth weight, suggesting that oral manifestations may reflect the overall systemic severity of maternal anemia and poor nutritional status. Therefore, careful oral examination during antenatal visits may aid in the early identification of women at higher risk of adverse maternal and fetal outcomes.

Clinical implications

The findings of the present study highlight the importance of early identification and management of maternal anemia during pregnancy. Routine antenatal Hb screening, nutritional counseling, iron and folic acid supplementation, and improved maternal healthcare access may facilitate the early detection and correction of maternal anemia. Public health interventions targeting women from lower socioeconomic groups and those with poor nutritional status are particularly important. Although the present findings demonstrate significant associations between maternal anemia and adverse birth outcomes, interventional studies are required to determine whether improving maternal anemia directly translates into improved maternal and neonatal outcomes. These results also emphasize the need for interdisciplinary collaboration among obstetricians, public health specialists, and dental healthcare professionals involved in maternal care and systemic health screening.

Limitations

The present study has certain limitations. First, it was conducted at a single tertiary care center, which may limit the generalizability of the findings to other populations. In addition, participants were recruited consecutively from antenatal clinics and were required to reside within the hospital catchment area to ensure adequate follow-up, which may have introduced selection bias. Second, serum ferritin and detailed micronutrient assessments were not performed in all participants, limiting the ability to distinguish iron-deficiency anemia from other causes of anemia. Third, dietary intake and long-term compliance with supplementation were assessed primarily through self-reporting, which may have introduced reporting bias. Fourth, the relatively small number of participants with severe anemia and the low frequency of stillbirths may have limited statistical power for rare outcomes and contributed to wider CIs for some estimates. Fifth, although all oral examinations were performed by a single examiner to ensure consistency, a formal assessment of examiner reliability was not undertaken. Finally, although multivariable regression analyses were performed, residual confounding from unmeasured factors, including maternal infections, inflammatory status, micronutrient deficiencies, and other biological variables not assessed in the present study, cannot be completely excluded. Furthermore, because oral mucosal changes and maternal anemia are biologically related, some overlap between these predictors may have influenced the observed associations. Therefore, the findings should be interpreted with appropriate caution and confirmed in larger multicenter studies incorporating comprehensive hematological, nutritional, and inflammatory assessments.

## Conclusions

The findings of the present study indicate that maternal anemia during pregnancy was significantly associated with adverse perinatal outcomes, particularly low birth weight, preterm birth, and stillbirth. A progressive increase in adverse neonatal outcomes was observed with increasing severity of anemia, demonstrating a significant dose-dependent relationship. Additionally, oral mucosal changes were markedly more prevalent among anemic women and were associated with unfavorable birth outcomes. Even after adjustment for potential confounding factors, maternal anemia remained independently associated with low birth weight. These findings support the importance of comprehensive antenatal evaluation, including early hematological and oral examination, and suggest that maternal anemia may serve as an important marker of increased risk for adverse neonatal outcomes. Further prospective and interventional studies are needed to confirm these findings and clarify causal relationships.
